# ‘*Lysinibacillus saudimassiliensis*’ sp. nov., a new bacterial species isolated from air samples in the urban environment of Makkah, Saudi Arabia

**DOI:** 10.1016/j.nmni.2016.12.011

**Published:** 2016-12-13

**Authors:** A. Papadioti, E.I. Azhar, F. Bibi, A. Jiman-Fatani, S.M. Aboushoushah, M. Yasir, D. Raoult, E. Angelakis

**Affiliations:** 1) Unité de Recherche sur les Maladies Infectieuses et Tropicales Emergentes: URMITE CNRS-IRD 198 UMR 6236, Aix Marseille Université, Faculté de Médecine, Marseille, France; 2) Special Infectious Agents Unit, King Fahd Medical Research Center, Jeddah, Saudi Arabia; 3) Department of Medical Laboratory Technology, Faculty of Applied Medical Sciences, Jeddah, Saudi Arabia; 4) Department of Medical Microbiology and Parasitology, Faculty of Medicine, King Abdulaziz University, Jeddah, Saudi Arabia

**Keywords:** Air isolates, culturomics, *Lysinibacillus saudimassiliensis*, new species, Saudi Arabia

## Abstract

We report here the main characteristics of ‘*Lysinibacillus saudimassiliensis*’ strain 13S34_air^τ^ (CSUR = P1222), a new species of the *Lysinibacillus* genus that was isolated from air samples in the city environment of Makkah, Saudi Arabia, during the pilgrim period of Hajj 2012.

As a part of a wider culturomics [1] and metagenomics study [2] in Saudi Arabia, we isolated a new bacterium, strain 13S34_air^Τ^, from two air samples in the urban environment of Makkah, Saudi Arabia, during the pilgrim period of Hajj 2012. For each air sample, a volume of 1 m^3^ was collected with a FCC-IV biological air sampler (AES Laboratories, Combourg, France) mounted with a nutrient agar plate containing the antifungal agent amphotericin (Majed Al-Buqami Co. BMC, Riyadh, Saudi Arabia) according to the manufacturer's instructions. No identification was obtained for the strain 13S34_air^Τ^ using our systematic matrix-assisted laser desorption/ionization time-of-flight mass spectrometry (MALDI-TOF MS) screening on a MicroFlex spectrometer (Bruker Daltonics, Bremen, Germany). Strain 13S34_air^Τ^ was cultured in 5% sheep's blood–enriched Columbia agar (bioMérieux, Marcy l'Etoile, France) for 2 days in an aerobic atmosphere at 37°C. Growth was observed only in aerobic conditions, and no growth occurred in anaerobic conditions. On Columbia agar, strain 13S34_air^Τ^ colonies were opaque and round, with a greyish color, and their size varied between 4 to 6 mm in diameter. The strain 13S34_air^Τ^ is a Gram-positive, obligate aerobic, endospore-forming, rod-shaped, catalase-positive and oxidase-negative bacterium. To test for spore formation, bacteria were heated at 80°C for 30 minutes and then were spread on blood-enriched Columbia agar. A positive result was taken after an overnight incubation at 37°C. Growth was observed in the range of 0.5 to 5% NaCl, with optimum growth at 0.5% NaCl. Observation under a light microscope showed bacterial motility.

The complete 16S rRNA gene was sequenced using fD1-rP2 primers as previously described and using a 3130-XL sequencer (Applied Biosciences, Saint Aubin, France) [3]. The strain 13S34_air^Τ^ exhibited a 96.4% sequence similarity with *Lysinibacillus sphaericus* (NR115724), which was the phylogenetically closest species with standing in nomenclature (Fig. 1). Consequently, it putatively classifies the strain 13S34_air^Τ^ as a new member of the genus *Lysinibacillus* within the family *Bacillaceae* in the phylum *Firmicutes*. The genus *Lysinibacillus* was first proposed in 2007 by Ahmed et al. [4] by the characterization of the type species *Lysinibacillus boronitolerans* and the transfer of *Bacillus fusiformis* to *Lysinibacillus fusiformis* comb. nov. and *Bacillus sphaericus* to *Lysinibacillus sphaericus* comb. nov. In 2012, an emended description of the genus *Lysinibacillus* was proposed by Jung *et al*. [5]. To date, the genus includes 21 species (http://www.bacterio.cict.fr/c/lysinibacillus.html); they are mostly environmental bacteria that are distributed primarily in soil [5].

Strain 13S34_air^Τ^ exhibited a 16S rRNA gene sequence divergence >1.3% with *L*. *sphaericus*, the closest related species with standing in nomenclature, which classifies it as a new representative of the *Lysinibacillus* genus isolated from air samples in the urban environment of Makkah. As a result, we propose the creation of ‘*Lysinibacillus saudimassiliensis*’ sp. nov., and the strain 13S34_air as the type strain.

## MALDI-TOF MS spectrum

The MALDI-TOF MS spectrum of 13S34_air^Τ^ is available online (http://www.mediterranee-infection.com/article.php?laref=256&titre=urms-database).

## Nucleotide sequence accession number

The 16S rRNA gene sequence of the strain 13S34_air^Τ^ was deposited in GenBank under accession number HG931343.1.

## Deposit in a culture collection

Strain 13S34_air^Τ^ was deposited in the Collection de Souches de l'Unité des Rickettsies (CSUR, WDCM 875) under number P1222.

## Figures and Tables

**Fig. 1:**
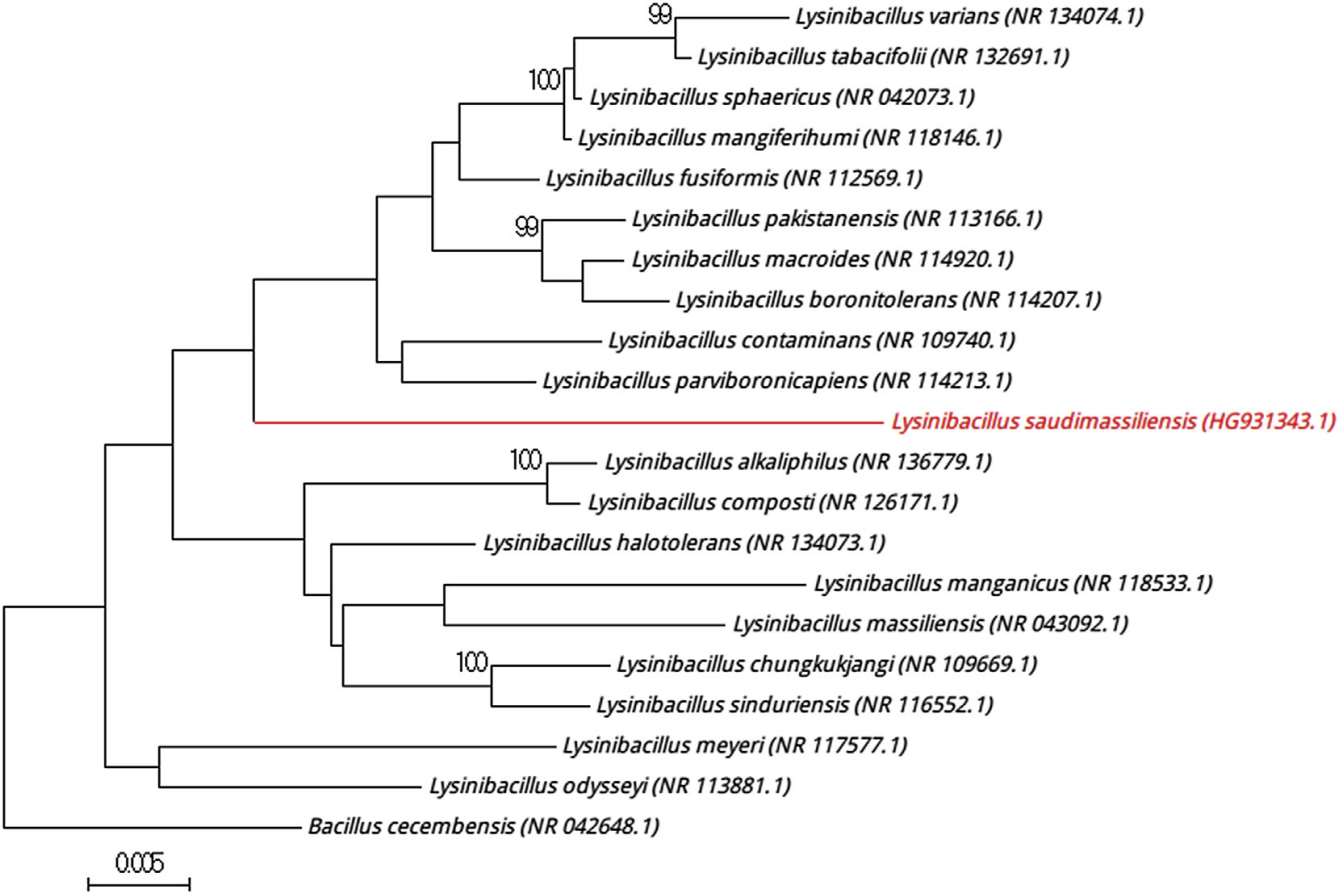
Phylogenetic tree highlighting position of ‘*Lysinibacillus saudimassiliensis*’ relative to other phylogenetically close members of *Lysinibacillus* genus. Numbers at nodes are percentages of bootstrap values obtained by repeating analysis 500 times to generate majority consensus tree. Only values >95% are displayed. Scale bar represents 0.5% nucleotide sequence divergence.
